# Incense Burning during Pregnancy and Birth Weight and Head Circumference among Term Births: The Taiwan Birth Cohort Study

**DOI:** 10.1289/ehp.1509922

**Published:** 2016-03-11

**Authors:** Le-Yu Chen, Christine Ho

**Affiliations:** 1 Institute of Economics, Academia Sinica, Taipei, Taiwan; 2 School of Economics, Singapore Management University, Singapore

## Abstract

**Background:**

Incense burning for rituals or religious purposes is an important tradition in many countries. However, incense smoke contains particulate matter and gas products such as carbon monoxide, sulfur, and nitrogen dioxide, which are potentially harmful to health.

**Objectives:**

We analyzed the relationship between prenatal incense burning and birth weight and head circumference at birth using the Taiwan Birth Cohort Study. We also analyzed whether the associations varied by sex and along the distribution of birth outcomes.

**Methods:**

We performed ordinary least squares (OLS) and quantile regressions analysis on a sample of 15,773 term births (> 37 gestational weeks; 8,216 boys and 7,557 girls) in Taiwan in 2005. The associations were estimated separately for boys and girls as well as for the population as a whole. We controlled extensively for factors that may be correlated with incense burning and birth weight and head circumference, such as parental religion, demographics, and health characteristics, as well as pregnancy-related variables.

**Results:**

Findings from fully adjusted OLS regressions indicated that exposure to incense was associated with lower birth weight in boys (–18 g; 95% CI: –36, –0.94) but not girls (1 g; 95% CI: –17, 19; interaction p-value = 0.31). Associations with head circumference were negative for boys (–0.95 mm; 95% CI: –1.8, –0.16) and girls (–0.71 mm; 95% CI: –1.5, 0.11; interaction p-values = 0.73). Quantile regression results suggested that the negative associations were larger among the lower quantiles of birth outcomes.

**Conclusions:**

OLS regressions showed that prenatal incense burning was associated with lower birth weight for boys and smaller head circumference for boys and girls. The associations were more pronounced among the lower quantiles of birth outcomes. Further research is necessary to confirm whether incense burning has differential effects by sex.

**Citation:**

Chen LY, Ho C. 2016. Incense burning during pregnancy and birth weight and head circumference among term births: The Taiwan Birth Cohort Study. Environ Health Perspect 124:1487–1492; http://dx.doi.org/10.1289/ehp.1509922

## Introduction

“Taiwan’s government on Friday urged the public to stop burning incense sticks and ritual money in honour of the dead … to better protect the environment” (Huffington Post 2010). Incense burning is a popular practice in Asian countries such as China, Hong Kong, Taiwan, Thailand, and Singapore, where traditional Chinese folk religion, Buddhism, and Taoism are mainstream religions (Friborg et al. 2008; Lin et al. 2008; Pan et al. 2014; Tse et al. 2011; Wang et al. 2011). It is also a common practice in countries such as India (Dewangan et al. 2013) and those in the Arabian Gulf (Cohen et al. 2013). At least 3,580 tons of incense are burned every year in temples in Taiwan, with the figure possibly doubling if incense burned at home is included (Lin et al. 2008). According to the Executive Yuan R.O.C. (Taiwan) (2014), “most Taiwanese visit a temple at a place and time of their choosing... people often burn incense sticks while praying to deities and burn spirit money. ... A vast number of people also have altars inside their homes, where they offer food, incense and prayers for their ancestors daily.”

It usually takes around 1–1.5 hr to burn a stick of incense, during which time, the incense stick emits smoke containing particulate matter (PM), gas products such as carbon monoxide (CO), sulfur dioxide (SO_2_), nitrogen dioxide (NO_2_), and volatile organic compounds (VOCs) such as benzene, toluene, and xylenes, which are potentially harmful to health (Lin et al. 2008). A few studies have studied the relationship between direct exposure to incense burning and health outcomes of adults and preschool or school age children. This literature has so far found that incense burning at home and in temples was related to an increase in the likelihood of respiratory health symptoms and allergies (Lee et al. 2003; Lin et al. 2008; Liu et al. 2013; Wang et al. 2011; Yang et al. 1997), and there is mixed evidence linking incense burning to atopic dermatitis and cancer (Chiang and Liao 2006; Lin et al. 2008; Wen et al. 2009).

To our knowledge, this study is the first that has analyzed the associations between prenatal incense burning and birth outcomes. Using data from the Taiwan Birth Cohort Study, we estimated the associations between prenatal incense burning and birth weight and head circumference at birth. Because incense smoke contains harmful components similar to those in cigarette smoke, we also estimated the associations between maternal smoking during pregnancy and birth outcomes. By allowing the associations between prenatal inputs and birth outcomes to differ by sex, we explored potential differences in associations between males and females. Finally, we analyzed how the associations varied across the birth outcomes’ distributions.

## Methods

### Data and Sample Construction

The Taiwan Birth Cohort Study (TBCS) is a longitudinal survey that follows 21,248 representatively sampled infants born in 2005 in Taiwan. The study was approved by the Institutional Review Board of Bureau of Health Promotion, the Department of Health and the Directorate-General of Budget, Accounting, and Statistics, Executive Yuan, ROC (No. 94-C3–0940005257), and informed consent was obtained from all participants. The study was conducted through face-to-face interviewer-administered questionnaires (Lung et al. 2011).

We used data from the first-wave interview, which contained information on pregnancy inputs, infant birth outcomes, parental health conditions before and during pregnancy, parental education, religion, and other demographics. Birth weight and head circumference variables were obtained from birth records, and the remaining variables used in our analysis were self-reported. We limited the sample to singleton (561 observations dropped) and full-term babies (> 37 gestational weeks; 1,206 observations dropped), whose parents were both 18–54 years of age (306 observations dropped), and whose parents were both Taiwanese (2,885 observations dropped). We dropped observations with missing values for birth weight (26 observations), incense burning (3 observations), maternal smoking during pregnancy (5 observations), maternal health indicators (58 observations), number of previous births (65 observations), parental weight and height (242 observations), parental chronic illness (108 observations), marital status (2 observations), and parental religion (8 observations). This left us with a sample of 15,773 infants among whom 8,216 were boys and 7,557 were girls.

### Outcome Variables and Main Explanatory Variables

We focused on two measures of infant health: birth weight (measured in grams) and head circumference at birth (measured in millimeters). We observed whether the household had a habit of burning incense from the question “Does your household have the habit of burning incense for religious purposes?” We constructed a dummy variable with a value of 1 if the household had the habit of burning incense and 0 otherwise. For the sample of all babies and the subsamples stratified by sex, we plotted the kernel densities (“kdensity”; Stata) for birth weight and head circumference across households that burned incense and households that did not. We also observed whether mothers smoked during pregnancy from the questions “Within the first three months of pregnancy (first trimester), did you smoke?” and “After the fourth month of pregnancy (second trimester), did you smoke?” We constructed a dummy variable taking a value of 1 if she smoked during pregnancy and 0 otherwise.

### Empirical Model and Control Variables

To estimate the associations between incense burning, maternal smoking and birth outcomes, we ran ordinary least squares (OLS) regressions (“reg”; Stata) as well as quantile regressions (“qreg”; Stata). The estimated coefficients of incense burning and maternal smoking from OLS were reported as forest plots (“coefplot”; Stata), and the estimated coefficients of incense burning from the quantile regressions were reported as quantile plots throughout the distribution of birth outcomes (“grqreg”; Stata). The OLS and quantile regressions were run separately for the outcomes of birth weight and head circumference, and separately on the full population of full-term births, the sample of boys, and the sample of girls. We also included an interaction term between incense burning during pregnancy and being a male baby in OLS regressions separately for each of the aforementioned outcomes on the full population of full term births. The *p*-value of the interaction term, from two-tailed *t*-tests, was obtained as regression output (“reg”; Stata).

To ensure that our estimates of interest are robust, we modeled each association using three different covariate specifications: model 1, adjusted for demographic variables only; model 2, additionally adjusted for parental health characteristics; and model 3, a fully adjusted model that also included pregnancy-related variables. Demographic characteristics included marital status (married, not married), mother’s and father’s ages at the time of birth (modeled using second-order polynomials), mother’s and father’s education (college graduate, yes/no), mother’s and father’s Chinese religion (Chinese folk religion, Buddhism, Taoism, or I-Kuan Tao, yes/no), and residence area (city, township, or town, modeled using indicator terms for each category). Parental health variables included mother’s prepregnancy body mass index (BMI) and father’s BMI (based on weight and height, modeled using second-order polynomials), maternal hepatitis B status (carrier, noncarrier), and any chronic illness in the mother or father (hypertension, cardiac disease, diabetes, asthma, atopic dermatitis, or allergic rhinitis; yes/no). Pregnancy-related variables included any maternal hospitalization during the pregnancy (yes/no), any pregnancy illness (severe morning sickness, fever infections, asthma attack, pregnancy induced hypertension, or toxemia of pregnancy; yes/no), gestational diabetes (yes/no), tocolysis (yes/no), any injury to the abdomen during pregnancy (yes/no), first pregnancy (yes/no), previous abortion (induced, yes/no), previous miscarriage (yes/no), any children < 6 years of age living in household (yes/no), and number of previous births (based on the total number of pregnancies minus the number of previous abortions, miscarriages, and stillbirths). The selected covariates were controlled for as they may be correlated with birth outcomes and incense burning.

We used an alpha of 5% as the significance level when reporting whether our estimates of interest are statistically significant.

## Results

Birth weight was observed for 15,773 babies (8,216 boys and 7,557 girls) in the sample, and head circumference at birth was observed for 14,488 babies (7,551 boys and 6,937 girls). The average birth weight for the population of full-term births, for boys, and for girls was 3,166 g, 3,217 g, and 3,111 g respectively. The average head circumference for the population of full-term births, for boys, and for girls was 334 mm, 336 mm, and 332 mm respectively.

Fifty-nine percent of households had the habit of burning incense and 3.6% of the mothers in the sample smoked during pregnancy. From kernel density graphs (see Figure S1), there was a slightly higher proportion of lower birth weight babies in households that burned incense compared with those that did not burn incense. The evidence was less conclusive for head circumference.

The average age of mothers and fathers was 29 and 32 years, respectively. Twenty-three percent of mothers and 28% of fathers were college educated. Approximately 77% of parents followed a Chinese religion such as Chinese folk religion, Buddhism, Taoism, and I-Kuan Tao. Thirty-six percent of mothers were experiencing a first pregnancy, and, on average, mothers had delivered 1.64 live births. Additional information on all variables used in our analysis are reported in Table 1.

**Table 1:** Study population characteristics*^a^* according to incense burning and child sex [*n* (%) or mean ± SD].

Characteristic	Whole sample	All		Boys		Girls	
		No incense	Burn incense	No incense	Burn incense	No incense	Burn incense
*n*	15,773	6,447	9,326	3,349	4,867	3,098	4,459
Child
Birth weight (g)	3166.0 ± 384.2	3176.1 ± 380.5	3159.1 ± 386.6*	3230.5 ± 377.4	3207.1 ± 384.9*	3117.3 ± 375.1	3106.7 ± 381.6
Head circumference (mm)^*b*^	334.0 ± 16.4	335.1 ± 16.3	333.3 ± 16.5*	337.3 ± 16.2	335.4 ± 16.6*	332.7 ± 16.1	331.05 ± 16.0*
Boy	8,216 (52)	3,349 (52)	4,867 (52)
Parental demographics
Mother smoked	569 (4)	247 (4)	322 (3)	127 (4)	173 (4)	120 (4)	149 (3)
Married	15,273 (97)	6,213 (96)	9,060 (97)*	3,242 (97)	4,725 (97)	2,971 (96)	4,335 (97)*
Mother’s age (years)	28.82 ± 4.59	29.72 ± 4.47	28.22 ± 4.57*	29.68 ± 4.47	28.31 ± 4.59*	29.77 ± 4.47	28.12 ± 4.55*
Father’s age (years)	31.55 ± 5.04	32.40 ± 4.96	30.97 ± 5.01*	32.34 ± 4.96	31.07 ± 4.96*	32.47 ± 4.97	30.85 ± 5.07*
Mother college	3,679 (23)	2,043 (32)	1,636 (18)*	1,076 (32)	843 (17)*	967 (31)	793 (18)*
Father college	4,374 (28)	2,428 (38)	1,946 (21)*	1,267 (38)	1,028 (21)*	1,161 (37)	918 (21)*
Mother Chinese religion^*c*^	12,098 (77)	4,227 (66)	7,871 (84)*	2,163 (65)	4,080 (84)*	2,064 (67)	3,791 (85)*
Father Chinese religion^*c*^	12,127 (77)	4,151 (64)	7,976 (86)*	2,130 (64)	4,151 (85)*	2,021 (65)	3,825 (86)*
Region—city	4,902 (31)	2,381 (37)	2,521 (27)*	1,234 (37)	1,292 (27)*	1,147 (37)	1,229 (28)*
Region—township	2,208 (14)	740 (11)	1,468 (16)*	369 (11)	750 (15)*	374 (12)	718 (16)*
Region—town	4,195 (27)	1,128 (17)	3,067 (32)*	3,349 (18)	1,629 (33)*	3,098 (17)	1,438 (32)*
Parental health
Mother’s BMI (kg/m^2^)	21.10 ± 3.20	21.00 ± 3.11	21.16 ± 3.26*	21.05 ± 3.09	21.16 ± 3.25	20.96 ± 3.14	21.15 ± 3.28*
Father’s BMI (kg/m^2^)	24.31 ± 3.45	24.38 ± 3.40	24.26 ± 3.48*	24.41 ± 3.40	24.30 ± 3.49	24.35 ± 3.40	24.21 ± 3.46
Mother, any chronic illness^*d*^	3,362 (21)	1,433 (22)	1,929 (21)*	773 (23)	1,030 (21)*	660 (21)	899 (20)
Father, any chronic illness^*d*^	3,599 (23)	1,583 (25)	2,016 (22)*	808 (24)	1,060 (22)*	775 (25)	956 (21)*
Mother hepatitis B	1,466 (9)	619 (10)	847 (9)	3,349 (9)	423 (9)	304 (10)	424 (10)
Prenancy-related variables
Any pregnancy hospitalization	990 (6)	364 (6)	626 (7)*	199 (6)	321 (7)	165 (5)	305 (7)*
Any pregnancy illness	4,489 (29)	1,824 (28)	2,265 (29)	897 (27)	1,326 (27)	927 (30)	1,339 (30)
Gestational diabetes	346 (2)	160 (2)	186 (2)	93 (3)	104 (2)	67 (2)	82 (2)
Tocolysis	3,744 (24)	1,519 (24)	2,225 (24)	798 (24)	1,154 (24)	721 (23)	1,071 (24)
Injury to abdomen	894 (6)	314 (5)	580 (6)*	177 (5)	277 (6)	137 (4)	303 (7)*
First pregnancy	5,681 (36)	2,340 (36)	3,341 (36)	1,207 (36)	1,708 (36)	1,133 (37)	1,633 (37)
Previous abortion	2,237 (14)	964 (15)	1,273 (14)*	520 (16)	706 (15)	444 (14)	567 (12)*
Previous miscarriage	1,535 (10)	648 (10)	887 (10)	345 (10)	465 (10)	303 (10)	422 (9)
Children < 6 years of age	6,290 (40)	2,392 (37)	3,898 (42)*	1,260 (37)	2,051 (42)*	1,132 (37)	1,847 (41)*
No. of live births	1.64 ± 0.75	1.61 ± 0.74	1.65 ± 0.76*	1.63 ± 0.76	1.67 ± 0.79*	1.60 ± 0.73	1.63 ± 0.73
^***a***^Birth weight and head circumference data were obtained from birth records linked to the TBCS. The remaining variables were self-reported. ^***b***^Head circumference was observed for 14,488 babies. ^***c***^Chinese religions include Chinese folk religion, Buddhism, Taoism, and I-Kuan Tao. ^***d***^Any chronic illness includes hypertension, cardiac disease, diabetes, asthma, atopic dermatitis, and allergic rhinitis. *p < 0.05 for *t*-test of the differences between households that burned incense and households that did not burn incense within each population group (all babies, boys, and girls).							

**Figure 1:**
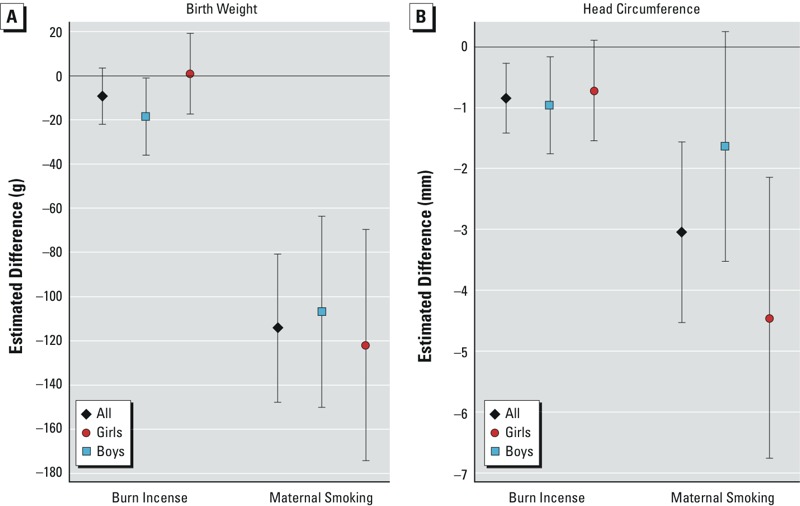
Associations between incense burning, smoking, and birth outcomes from OLS. Estimated effects of incense burning and smoking on birth outcomes were analyzed with OLS regressions. We controlled for demographic variables (marital status, mother’s age, education, and religion, father’s age, education, and religion, region of birth), parental health characteristics (mother’s BMI and chronic illness, father’s BMI and chronic illness), and pregnancy-related variables (hospitalization, pregnancy illness, gestational diabetes, tocolysis, injury to abdomen, first pregnancy, previous abortion, previous miscarriage, children < 6 years of age, number of live births). Population of singleton term babies (*n* = 15,773), boys (*n* = 8,216), and girls (*n* = 7,557). Head circumference regressions: population of singleton term babies (*n* = 14,488), boys (*n* = 7,551), and girls (*n* = 6,937). 95% confidence intervals are illustrated. Corresponding numeric data are provided in Tables S1 and S2.

The OLS regression results for the outcomes of birth weight and head circumference at birth are reported in Figure 1. We carried out estimations based on the sample of all babies as well as on the boy and girl subsamples. Within each sample and subsample, we report results for the full covariate specification including demographic variables, parental health characteristics, and pregnancy-related variables. The point estimates were qualitatively similar across all covariate specifications (see Tables S1 and S2).

There was a negative but nonsignificant association (*p* > 0.05) between incense burning and birth weight in the overall sample based on the fully adjusted model [–9.18 g; 95% confidence interval (CI): –21.8, 3.4] (Figure 1; see also Table S1). However, incense burning was associated with significantly lower birth weight in boys (–18.4 g; 95% CI: –36, –0.94), whereas there was no association in girls (1.0 g; 95% CI: –17.2, 19.3; interaction *p*-value = 0.31). There was also a negative association between incense burning and average head circumference at birth. Incense burning was associated significantly with smaller head circumference in the full sample of babies (–0.84 mm; 95% CI: –1.41, –0.27) and in boys (–0.95 mm; 95% CI: –1.75, –0.16). The associations were negative but nonsignificant in girls (–0.71 mm; 95% CI: –1.54, 0.11; interaction *p*-value = 0.73). The interaction *p*-value from the head circumference regression was larger than that from the birth weight regression, suggesting that sex differences need to be confirmed through future research.

**Figure 2:**
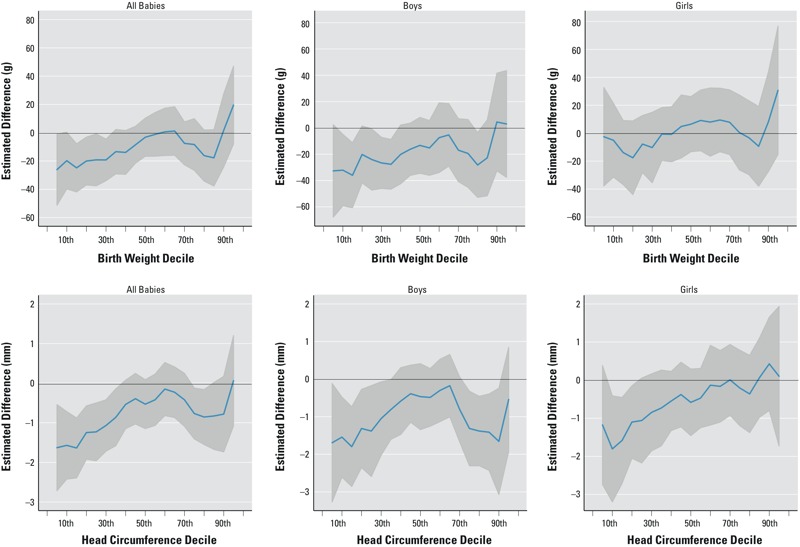
Quantile plots for birth weight (top) and head circumference (bottom) deciles. Estimated effects of incense burning by quantile and 95% CI using quantile plots. The 10th, 30th, 50th, 70th, and 90th deciles are shown on the horizontal axes. We controlled for demographic variables (marital status, mother’s age, education and religion, father’s age, education, and religion, region of birth), parental health characteristics (mother’s BMI and chronic illness, father’s BMI and chronic illness), and pregnancy-related variables (hospitalization, pregnancy illness, gestational diabetes, tocolysis, injury to abdomen, first pregnancy, previous abortion, previous miscarriage, children < 6 years of age, number of live births). Population of singleton term babies (*n* = 15,773), boys (*n* = 8,216), and girls (*n* = 7,557). Head circumference plots: population of singleton term babies (*n* = 14,488), boys (*n* = 7,551), and girls (*n* = 6,937). Corresponding numeric data are provided in Tables S3 and S4.

The quantile regression results of the effects of incense burning on the outcomes of birth weight and head circumference are summarized in Figure 2 (see Tables S3 and S4 for corresponding data). The plot was estimated for the entire distribution of birth outcomes. We indicate the 10th, 30th, 50th, 70th, and 90th deciles on the horizontal axes. Regarding the birth weight results, Figure 2 indicates that the estimated coefficients for incense burning were negative for the overall and the boy samples across the 10th to 70th deciles. For the boy sample, these estimates across the 10th to 30th deciles were also significant. In particular, incense burning was associated with a decrease of 26–32 g across the 10th to 30th deciles for boys. Thereafter, the associations between incense burning and birth weight were statistically insignificant for both the overall and boy samples. For the girl subsample, these coefficient estimates were negative for the 10th and 30th deciles but statistically insignificant.

Meanwhile, for the outcome of head circumference at birth, the estimates were negative across all deciles for the samples of all babies and boys (Figure 2; see also Table S4). Among those two samples, the estimated coefficients for incense burning were significant for the 10th to 30th deciles. The point estimates for those deciles ranged from –1 to –1.5 mm for all babies and for boys. For the boy subsample, the associations were significant for the upper 70th to 90th deciles as well, with a decrease of around 0.8 to 1.7 mm in head circumference. On the other hand, the associations between incense burning and head circumference for girls were negative and significant only for the 10th decile (1.88 mm; 95% CI: –3.18, –0.43).

The results concerning maternal smoking during pregnancy show that the estimated coefficients for maternal smoking were all negative and generally significant in the mean regressions for both outcomes. As can be seen in Figure 1, maternal smoking was associated with decrease in birth weight of 114 g (95% CI: –148, –81) in the full sample, 107 g (95% CI: –150, –63) for boys, and 122 g (95% CI: –175, –70) for girls. Similarly, maternal smoking was associated with a decrease in head circumference of 3 mm (95% CI: –4.5, –1.6) for all babies, 1.6 mm (95% CI: –3.5, 0.25) for boys, and 4 mm for girls (95% CI: –6.8, –2.1). The associations were also negative in the quantile regressions.

## Discussions

We found that the associations between incense burning and birth outcomes were generally negative, had smaller *p*-values for the outcome of head circumference at birth than that of birth weight, and were more evident for the lower than for the upper quantiles of the outcome distribution. Although there is some indication that the associations may have been more pronounced for boys in the birth weight regression, further research is required to confirm sex differences. Poor infant health at birth, as measured by birth weight and head circumference, has been widely associated with poorer long-term outcomes such as latent health issues, lower educational attainment, and adverse labor market outcomes (Behrman and Rosenzweig 2004; Black et al. 2007; Boardman et al. 2002; Case et al. 2005; Chatterji et al. 2014; Currie and Hyson 1999; Gale et al. 2004; Gross et al. 1978, 1983; Stathis et al. 1999). In particular, low birth weight and small head circumference have been associated with cognitive and neurological impairments and poorer health as adults (Behrman and Rosenzweig 2004). Our findings therefore suggest that incense burning might also be harmful to health in the long term.

Some nonmonotonicity across quantiles seemed to be present in the boy and the full samples for the outcomes of both birth weight and head circumference, with the absolute magnitudes and significance of the estimates initially decreasing as we moved from the lowest to the middle quantiles and then increasing around the 70th decile. This may indicate that both the smallest and largest babies at the two extreme of the distributions of birth outcomes were more vulnerable to incense burning, whereas babies of average size were less vulnerable to incense burning. Such nonmonotonicities were coherent with previous evidence of nonmonotonicities in the smoking literature (Abrevaya and Dahl 2008; Bache et al. 2013). In addition, our results on the boy and girl subsamples seemed to indicate the relative vulnerability of male fetuses to incense burning compared to that of female fetuses.

The magnitudes of our significant estimates of the associations with maternal smoking, which ranged from –84 g to –155 g in the birth weight regressions (see Tables S1 and S3), were within the range of –50 g to –600 g found in the literature on maternal smoking (yes/no) and birth weight (Abrevaya 2006; Abrevaya and Dahl 2008; Lindley et al. 2000; Reichman et al. 2009). On the other hand, the magnitudes of our significant estimates of the associations with maternal smoking, which ranged from –2.2 mm to –4.5 mm in the head circumference regressions (see Tables S2 and S4), were inclusive of the range of –3 mm to –4 mm found in the study by Lindley et al. (2000), who studied light maternal smoking (1–9 cigarettes a day, yes/no) and heavy maternal smoking (> 10 cigarettes a day, yes/no). We also noted that the coefficient estimates for maternal smoking were larger in magnitude and yielded more significant results than those for incense burning in most of the regressions. Such a finding was not surprising because cigarette smoking incurred direct inhalation of harmful chemicals by the pregnant mother, whereas the incense burning was a more passive form of inhalation.

The various potentially harmful chemical components in incense smoke, such as PM, CO, SO_2_, NO_2_, and VOCs, are also present in cigarette smoke (Lin et al. 2008). It has been reported that maternal smoking during pregnancy led to lower birth weight (Abrevaya 2006; Abrevaya and Dahl 2008; Bharadwaj et al. 2014; Del Bono et al. 2012; Reichman et al. 2009; Rosenzweig and Schultz 1983) and smaller head circumference (Andersen et al. 2009; Källén 2000; Kayemba-Kay’s et al. 2008; Lindley et al. 2000). Similarly, the literature has documented that there was a negative association between air pollution and birth weight (Bell et al. 2007; Brauer et al. 2008; Currie et al. 2009; Currie and Walker 2011; Liu et al. 2003; Salam et al. 2005) as well as head circumference at birth (Choi et al. 2006).

Although there is an emerging literature relating direct exposure to incense burning and adults’ health or preschool- and school-age children’s health (Friborg et al. 2008; Lin et al. 2008; Pan et al. 2014; Tse et al. 2011; Wang et al. 2011), to our knowledge, our study is the first to provide evidence of the associations between prenatal incense burning and birth outcomes. Given the relative popularity of incense burning traditions in many countries, and given the presence of potentially harmful ingredients in incense smoke, it is important to understand how incense burning during pregnancy relates to infants’ health outcomes at birth.

Our rich data set allowed us to control extensively for factors that may be correlated with incense burning and birth outcomes, such as parental religion and demographic characteristics of the family, parental health endowments and maternal pregnancy-related variables. Our large sample size (15,773 observations) also allowed us to conduct the analysis separately by sex, which seemed to suggest some differences between girls and boys for the outcome of birth weight; however, further research is necessary to confirm sex differences. We also performed mean and quantile regressions, which allowed us to analyze whether the associations differed in means and distributions across birth outcomes.

Whereas our rich data set enabled us to estimate the associations between incense burning practice and birth outcomes, we note the following limitations of our study: First, there may be other determinants of birth outcomes that we could not control for due to data limitations. For instance, season of birth is known to affect birth outcomes (Currie and Schwandt 2013), so controlling for birth month may help improve the precision of our estimates. Most variables were self-reported, and there were also fewer girls than boys in the sample. In addition, we did not observe whether the pregnant mother was always present during the incense burning ritual. Moreover, we did not observe whether the incense was being specifically burnt at home or at temples, nor the degree of ventilation in the room. We also did not observe the amount of incense being burnt nor exposure to paternal smoking. We thus did not have information on the exact amount of secondhand smoke exposure. Nevertheless, given the prevalence of incense burning practice in many countries, we believe that future research on estimating the relationship between the potential amounts of incense inhaled and birth outcomes may have important policy implications on the incense-burning habits of households.

## Conclusions

There is a growing interest on the relationship between ambient air and health outcomes. In this study, we addressed the issues of incense smoke and infant birth outcomes. Using a nationwide data set from the Taiwan Birth Cohort Study, we estimated the empirical relationship between incense burning during pregnancy, birth weight, and head circumference at birth. Our findings from mean and quantile regressions suggest that the associations between incense burning and infant birth outcomes were generally negative. Moreover, the associations with lower quantiles of the distributions of birth outcomes appeared to be more pronounced than those with the upper quantiles. Our findings need to be confirmed in other populations, including differences between boys and girls.

## Supplemental Material

(336 KB) PDF
