# Supplementary material for: Incense Burning during Pregnancy and Birth Weight and Head Circumference among Term Births: The Taiwan Birth Cohort Study
Source: Environ Health Perspect. 2016 Mar 11;124(9):1487–92. doi: 10.1289/ehp.1509922 (PMC5010412; doi:10.1289/ehp.1509922)
Supplement: (336 KB) PDF [file ehp.1509922.s001.acco.pdf]

Environ Health Perspect
DOI: 10.1289/ehp.1509922

Note to readers with disabilities: *EHP* strives to ensure that all journal content is accessible to all readers. However, some figures and Supplemental Material published in *EHP* articles may not conform to 508 standards due to the complexity of the information being presented. If you need assistance accessing journal content, please contact ehp508@niehs.nih.gov. Our staff will work with you to assess and meet your accessibility needs within 3 working days.

Supplemental Material

Incense Burning during Pregnancy and Birth Weight and Head Circumference among Term Births: The Taiwan Birth Cohort Study

Le-Yu Chen and Christine Ho

Table of Contents

Table S1: Incense Burning and Birth Weight
Table S2: Incense Burning and Head Circumference
Table S3: Incense Burning and Birth Weight Quantile Estimates
Table S4: Incense Burning and Head Circ. Quantile Estimates
Figure S1: Kernel Density Plots. Note: Estimates are not adjusted for any covariates and are based on full-term singleton infants. Birth weight: all babies (n = 15,773), boys (n = 8,216) and girls (n = 7,557). Head circumference sample: all babies (n = 14,488), boys (n = 7,551) and girls (n = 6,937).

DOI: 10.1289/ehp.1509922

Table S1: Incense Burning and Birth Weight[a]

Exposure	Model 1 β (95% CI)	Model 2 β (95% CI)	Model 3 β (95% CI)
**All Babies** (n = 15,773)			
Burn incense	-7.17 (-19.98, 5.65)	-9.55 (-22.15, 3.06)	-9.18 (-21.78, 3.43)
Maternal smoking	-112.4** (-146.4,-78.44)	-106.3** (-139.6,-73.13)	-114.2** (-147.9,-80.59)
R-squared	0.04	0.07	0.08
**Boys** (n = 8,216)			
Burn incense	-18.25* (-36.01, -0.49)	-18.83* (-36.33,-1.33)	-18.41* (-35.88, -0.94)
Maternal smoking	-102.1** (-145.9,-58.33)	-99.06** (-141.6,-56.50)	-106.8** (-150.0,-63.49)
R-squared	0.02	0.05	0.06
**Girls** (n = 7,557)			
Burn incense	4.65 (-13.89, 23.20)	0.13 (-18.08, 18.33)	1.02 (-17.21,19.26)
Maternal smoking	-125.5** (-178.2,-72.78)	-114.5** (-166.1,-62.82)	-122.1** (-174.5,-69.63)
R-squared	0.02	0.06	0.06

[a]Results from ordinary least square regressions. 95% Confidence intervals reported in parentheses. Model 1 controls for demographic variables (marital status, mother's age, education and religion, father's age, education and religion, region of birth). Model 2 is additionally adjusted for parental health characteristics (mother's bmi and chronic illness, father's bmi and chronic illness). Model 3 is additionally adjusted for parental health characteristics and pregnancy related variables (hospitalized, pregnancy illness, gestational diabetes, tocolysis, injury to abdomen, first pregnancy, previous abortion, previous miscarriage, children below 6, no. of live births).

*p < .05; **p < .01.

DOI: 10.1289/ehp.1509922

Table S2: Incense Burning and Head Circumference[a]

Exposure	Model 1 β (95% CI)	Model 2 β (95% CI)	Model 3 β (95% CI)
**All Babies** (n = 14,488)			
Burn incense	-0.74* (-1.31,-0.17)	-0.80** (-1.37,-0.23)	-0.84** (-1.41,-0.27)
Maternal smoking	-2.84** (-4.32,-1.36)	-2.70** (-4.17,-1.23)	-3.04** (-4.52,-1.56)
R-squared	0.05	0.05	0.05
**Boys** (n = 7,551)			
Burn incense	-0.95* (-1.75,-0.15)	-0.96* (-1.76,-0.17)	-0.95* (-1.75,-0.16)
Maternal smoking	-1.27 (-3.14, 0.61)	-1.22 (-3.07, 0.64)	-1.63† (-3.51, 0.25)
R-squared	0.03	0.04	0.04
**Girls** (n = 6,937)			
Burn incense	-0.49 (-1.32, 0.33)	-0.62 (-1.45, 0.20)	-0.71† (-1.54, 0.11)
Maternal smoking	-4.55** (-6.85,-2.26)	-4.24** (-6.52,-1.97)	-4.44** (-6.75,-2.14)
R-squared	0.03	0.05	0.06

[a]Results from ordinary least square regressions. 95% Confidence intervals reported in parentheses. Model 1 controls for demographic variables (marital status, mother's age, education and religion, father's age, education and religion, region of birth). Model 2 is additionally adjusted for parental health characteristics (mother's bmi and chronic illness, father's bmi and chronic illness). Model 3 is additionally adjusted for parental health characteristics and pregnancy related variables (hospitalized, pregnancy illness, gestational diabetes, tocolysis, injury to abdomen, first pregnancy, previous abortion, previous miscarriage, children below 6, no. of live births).

†p < .10; *p < .05; **p < .01.

Environ Health Perspect
DOI: 10.1289/ehp.1509922

Table S3: Incense Burning and Birth Weight Quantile Estimates[a]

Exposure	10th Decile<br>β (95% CI)	30th Decile<br>β (95% CI)	50th Decile<br>β (95% CI)	70th Decile<br>β (95% CI)	90th Decile<br>β (95% CI)
**All Babies** (n = 15,773)					
Burn incense	-19.73† (-39.75, 0.30)	-19.21* (-34.03,-4.39)	-3.19 (-17.62, 11.24)	-7.51 (-23.81, 8.79)	1.704 (-24.47, 27.88)
Maternal smoking	-155.3** (-206.2,-104.4)	-93.11** (-130.8,-55.4)	-89.47** (-126.2,-52.77)	-116.0** (-157.5,-74.59)	-99.11** (-165.7,-32.53)
Average birthweight	2,700 g	2,972 g	3,150 g	3,350 g	3,650 g
**Boys** (n = 8,216)					
Burn incense	-32.09* (-59.93, -4.24)	-26.59* (-46.83,-6.35)	-13.26 (-34.10, 7.58)	-16.91 (-40.08, 6.27)	4.52 (-32.63, 41.67)
Maternal smoking	-125.9** (-195.9,-55.85)	-93.52** (-144.5,-42.59)	-86.12** (-138.6,-33.68)	-110.6** (-168.9,-52.33)	-84.22† (-177.7, 9.24)
Average birthweight	2,750 g	3,005 g	3,200 g	3,400 g	3,700 g
**Girls** (n = 7,557)					
Burn incense	-5.07 (-33.89, 23.75)	-10.27 (-32.12, 11.58)	6.45 (-13.83, 26.73)	7.85 (-15.45, 31.14)	8.05 (-30.05, 46.14)
Maternal smoking	-155.6** (-230.0,-81.12)	-105.3** (-161.8,-48.91)	-83.95** (-136.3,-31.58)	-106.7** (-166.8,-46.53)	-66.13 (-164.5, 32.23)
Average birthweight	2,658 g	2,910 g	3,100 g	2,390 g	3,600 g

[a]Results from quantile regressions. 95% Confidence intervals reported in parentheses. All regressions include controls for parental demographics (marital status, mother's age, education and religion, father's age, education and religion, region of birth), parental health characteristics (mother's bmi and chronic illness, father's bmi and chronic illness) and maternal pregnancy related issues (hospitalized, pregnancy illness, gestational diabetes, tocolysis, injury to abdomen, first pregnancy, previous abortion, previous miscarriage, children below 6, no. of live births).

†$p < .10$; *$p < .05$; **$p < .01$.

Table S4: Incense Burning and Head Circumference Quantile Estimates[a]

Exposure	10th Decile β (95% CI)	30th Decile β (95% CI)	50th Decile β (95% CI)	70th Decile β (95% CI)	90th Decile β (95% CI)
**All Babies** (n = 14,488)					
Burn incense	-1.58** (-2.47,-0.67)	-1.07** (-1.80,-0.34)	-0.53† (-1.16, 0.09)	-0.42 (-1.12, 0.28)	-0.78 (-1.72, 0.15)
Maternal smoking	-3.82** (-6.12,-1.52)	-2.78** (-4.64,-0.92)	-2.20** (-3.80,-0.60)	-1.22 (-3.33, 0.25)	-2.35† (-4.74, 0.04)
Average head circumference	315 mm	325 mm	330 mm	340 mm	350 mm
**Boys** (n = 7,551)					
Burn incense	-1.54** (-2.64,-0.45)	-1.05* (-2.00,-0.10)	-0.47 (-1.41, 0.47)	-0.81** (-1.79, 0.17)	-1.66* (-3.15,-0.16)
Maternal smoking	-2.08 (-4.86, 0.71)	-1.88 (-4.31,-0.54)	-0.36 (-2.75, 2.04)	-0.26 (-2.76, 2.23)	-1.37 (-5.17, 2.43)
Average head circumference	320 mm	330 mm	335 mm	345 mm	355 mm
**Girls** (n = 6,937)					
Burn incense	-1.81** (-3.18,-0.43)	-0.85 (-1.94, 0.24)	-0.58 (-0.58, 0.24)	0.01 (-0.93, 0.95)	0.43 (-0.83, 1.69)
Maternal smoking	-4.29* (-7.80,-0.72)	-3.67* (-6.48,-0.86)	-2.50* (-4.62,-0.38)	-3.82** (-6.24,-1.40)	-3.31* (-6.56,-0.06)
Average head circumference	310 mm	325 mm	330 mm	340 mm	350 mm

[a]Results from quantile regressions. 95% Confidence intervals reported in parentheses. All regressions include controls for parental demographics (marital status, mother's age, education and religion, father's age, education and religion, region of birth), parental health characteristics (mother's bmi and chronic illness, father's bmi and chronic illness) and maternal pregnancy related issues (hospitalized, pregnancy illness, gestational diabetes, tocolysis, injury to abdomen, first pregnancy, previous abortion, previous miscarriage, children below 6, no. of live births).

†p < .10; *p < .05; **p < .01.

Environ Health Perspect
DOI: 10.1289/ehp.1509922

Figure S1: Kernel Density Plots

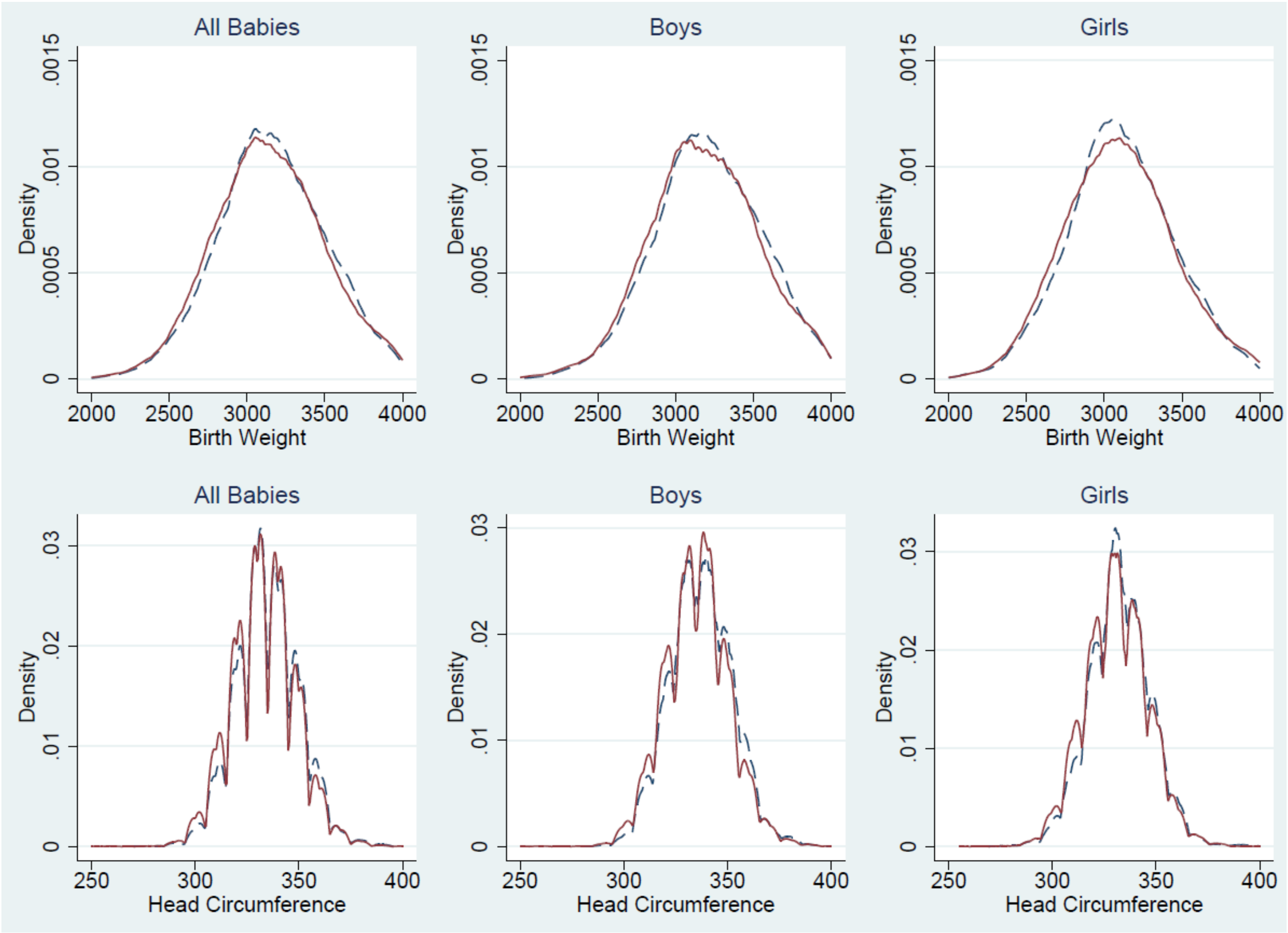

Legend: - - - - - - - - No Incense ——————— Burn Incense

Note: Estimates are not adjusted for any covariates and are based on full-term singleton infants. Birth weight: all babies (n = 15,773), boys (n = 8,216) and girls (n = 7,557). Head circumference sample: all babies (n = 14,488), boys (n = 7,551) and girls (n = 6,937).
